# Supplementary material for: Cell kinetics of auxin transport and activity in Arabidopsis root growth and skewing
Source: Nat Commun. 2021 Mar 12;12:1657. doi: 10.1038/s41467-021-21802-3 (PMC7954861; doi:10.1038/s41467-021-21802-3)
Supplement: Supplementary file 1 — Supplementary Information [file 41467_2021_21802_MOESM1_ESM.pdf]

Cell kinetics of auxin transport and activity in *Arabidopsis* root growth and skewing

Hu et al. 2021

Supplemental Data

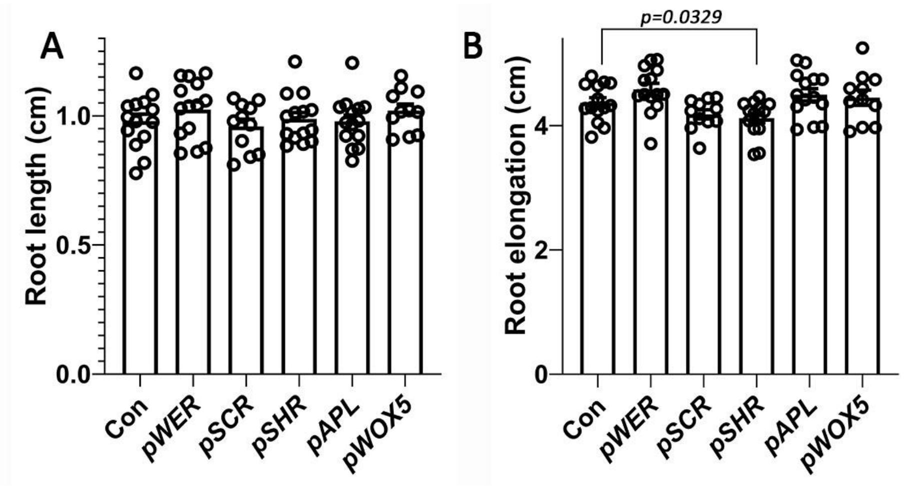

Supplementary Fig. 1. Cell-type-specific *YUC1-TAA1* inducible lines are not leaky. Root length and growth of cell-type-specific *YUC1-TAA1* inducible lines without estradiol treatment. **(a)** root length at day 4. **(b)** Root elongation between day 4 to day 11. Controls (Ctr) are *DR5:VENUS* seedlings. Significant at $p < 0.05$ (*), two-tailed t-test, (n ≥ 11; n = 14 for Ctr, *pWER*, *pAPL* and *pSHR*, n =11 for *pSCR* and *pWOX5*). Columns and error bars represent the means ± SE.

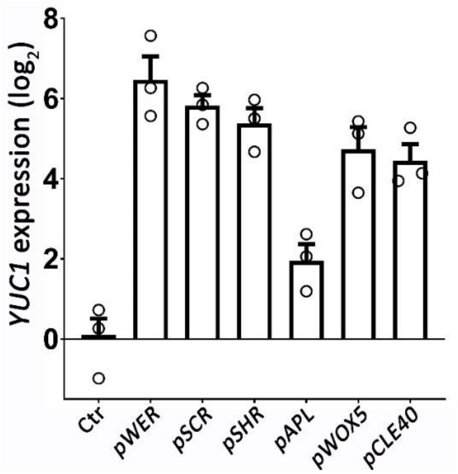

Supplementary Fig. 2. Relative expression level of *YUC1* following cell-type-specific estradiol induction. The expression level of *YUC1* was measured after 90-min estradiol treatment (5 µM). mRNA levels were assayed by quantitative RT-PCR. Control (Ctr) is Col-0 seedlings. Shown are means ± SE (n = 3).

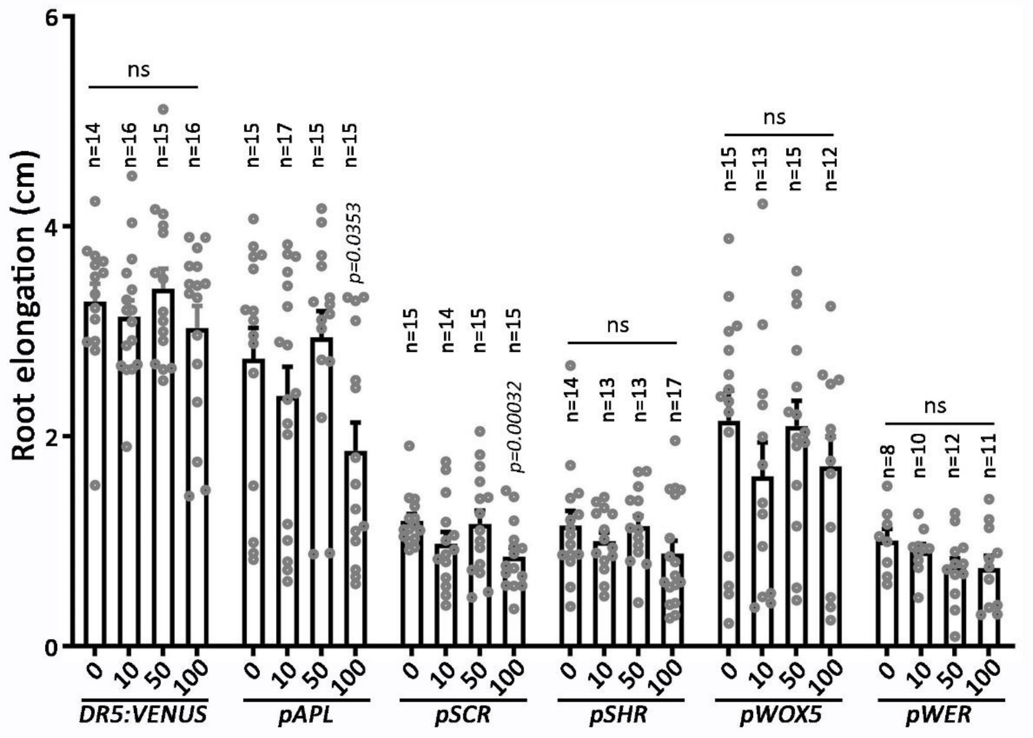

Supplementary Fig. 3. L-tryptophan effect on cell-type-specific IAA-mediated root growth. The cell-type-specific IAA inducible lines were grown on MS for 4 days and then transferred to plates containing different concentration of L-tryptophan in the presence of 5 µM estradiol. Root elongation between day 4 to day 10 was recorded. Shown are means ± SE. Significant at $P < 0.05$(*), two-tailed t-test. n number is indicated for each line and treatment. *DR5:VENUS* are control plants as all IAA inducible lines are in the *DR5:VENUS* background. ns indicates for not significant.

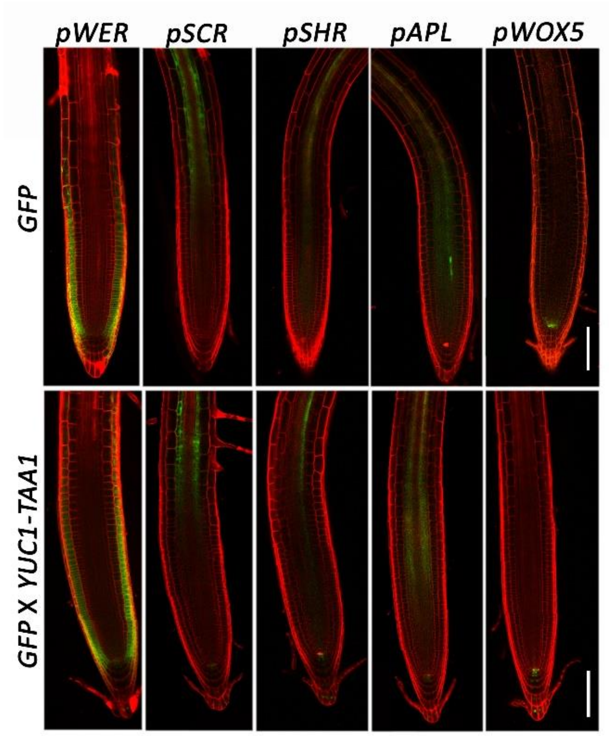

Supplementary Fig. 4. *YUC1-TAA1* lines with cell-type-specific inducible promoters do not expand transcriptionally following 6 h of estradiol treatment. Top: Expression pattern of cell-type-specific promoters after 6-h estradiol treatment (5 µM). **Bottom**: Expression pattern of the same promoters in the background of cell-type-specific *YUC1-TAA1*. F1 seeds were generated by crossing homozygous parents (cell-type-specific GFP lines with cell-type-specific *YUC1-TAA1; DR5:VENUS* lines). *pWOX5* is *pWOX5:mCHERRY*. The *DR5:VENUS* channel was imaged in a separate track not shown here. The experiment was independently repeated three times. Scale bar = 100 µm.

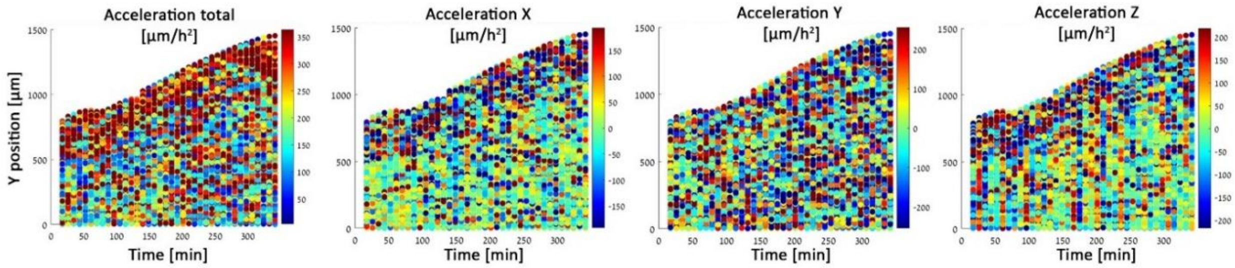

Supplementary Fig. 5. Acceleration map over time of control *pWOX5:XVE:YUC1-TAA1; DR5:VENUS; 35S:H2B-RFP* roots without estradiol treatment. Root tip is positioned upwards. Data are a concatenate of three independent root movies, each with ~1000 nuclei. On the color scale, red indicates high velocity, and blue indicates low velocity.

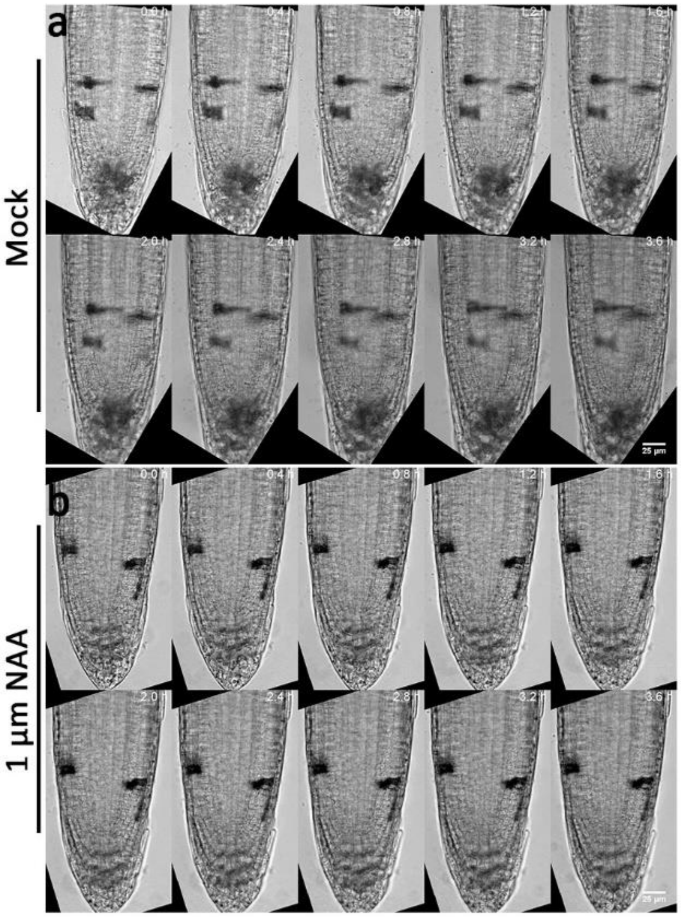

Supplementary Fig. 6. Monitoring auxin-dependent root skewing dynamics using long-term vertical-stage microscopy of UV laser ablation sites. Images of **a)** mock-treated and **b)** 1 µm NAA-treated roots are shown. Time in hours is shown at the top. Black spots indicate for laser ablated cells. The experiment was independently repeated three times. Scale bar = 25 µm. The 19-h movie is provided as Supplementary Movie 4.

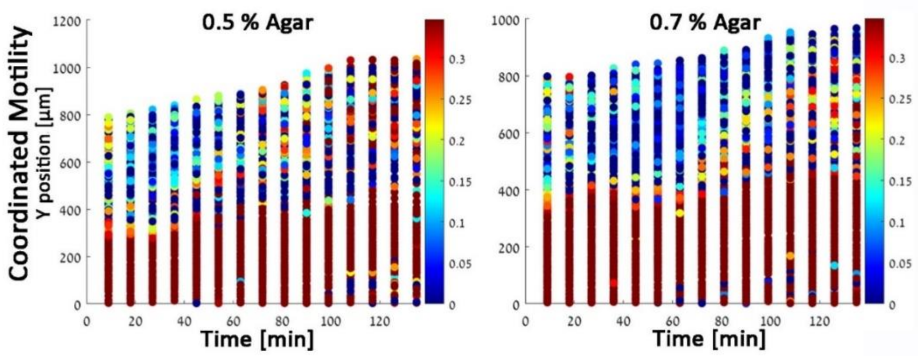

Supplementary Fig. 7. Root skewing is not affected by half-liquid medium. *pWOX5:XVE:YUC1-TAA1; DR5:VENUS; 35S:H2B-RFP* seedlings were mounted in 0.5% or 0.7% MS-agar. Root tip faces upwards as shown in Fig. 2. T_0 indicates 20 min following mock treatment. On the color scale, red indicates high coordinated motility and blue indicates low coordinated motility.

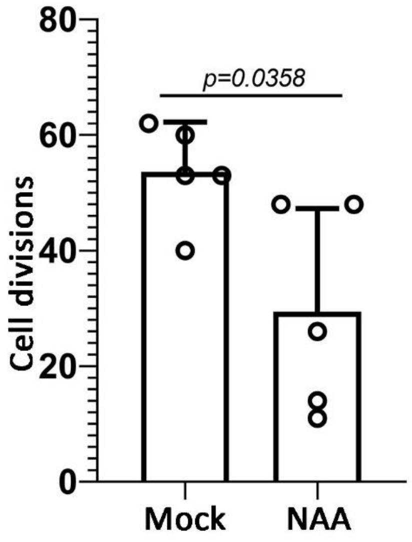

Supplementary Fig. 8. NAA effect on cell divisions during root growth. Shown are cell-division frequencies in the root meristem zone treated with vehicle (mock) or 1 µM NAA determined using 20-h time course vertical-stage microscopy. Shown are means ± SD, two-tailed t-test (n = 5).

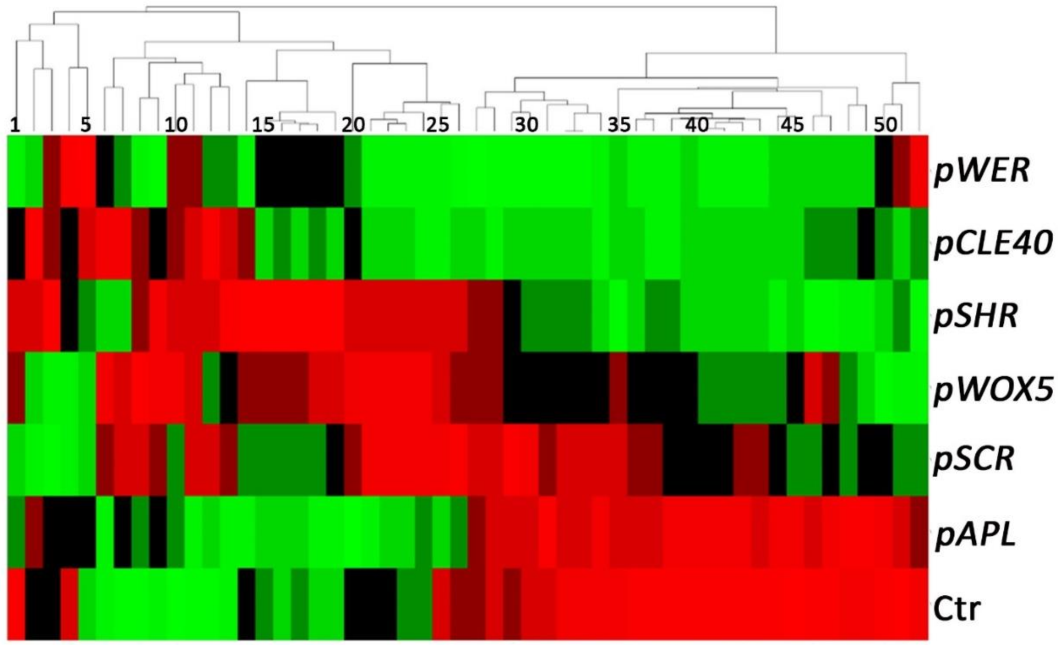

Supplementary Fig. 9. Hierarchical clustergram analysis of the indicated cell-type-specific *YUC1-TAA1* inducible lines grouped by morphokinetic parameters. Ctr indicates control mock-treated *pWOX5:XVE:YUC1-TAA1; DR5:VENUS* seedlings. This image is shown in Fig. 3C; here, the numbers at the top correspond to the parameter number (No.) in **Supplementary Table 1**. Data were concatenated from three independent root movies for each line, each with ~1000 nuclei. T_0 is 20 min following estradiol treatment.

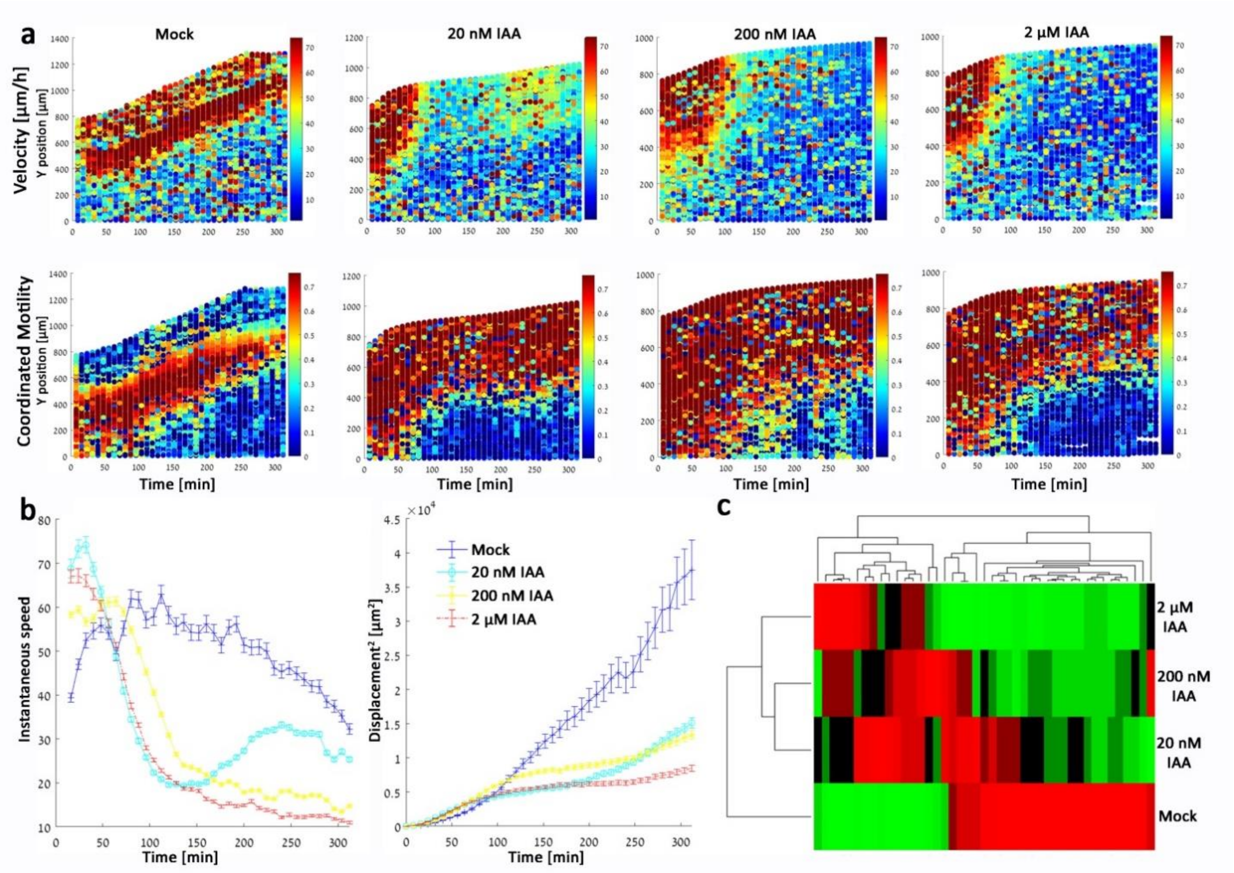

Supplementary Fig. 10. *Arabidopsis* root kinetics in response to increasing IAA concentrations. **(a)** Single-cell velocity (top) and coordinated motility (bottom) maps following treatments with different concentrations of IAA. **(b)** Instantaneous speed and cell displacement over time. Shown are means (±SD), n ≥ 300 nuclei. The n number for each treatment and time point is indicated at Supplementary Source data file. **(c)** Hierarchical clustergram analysis based on morphokinetic parameters of the plants treated with the indicated concentrations of IAA. T_0 indicates 20 min after IAA treatment. Mock indicates vehicle-treated *35S*:*H2B-RFP* seedlings.

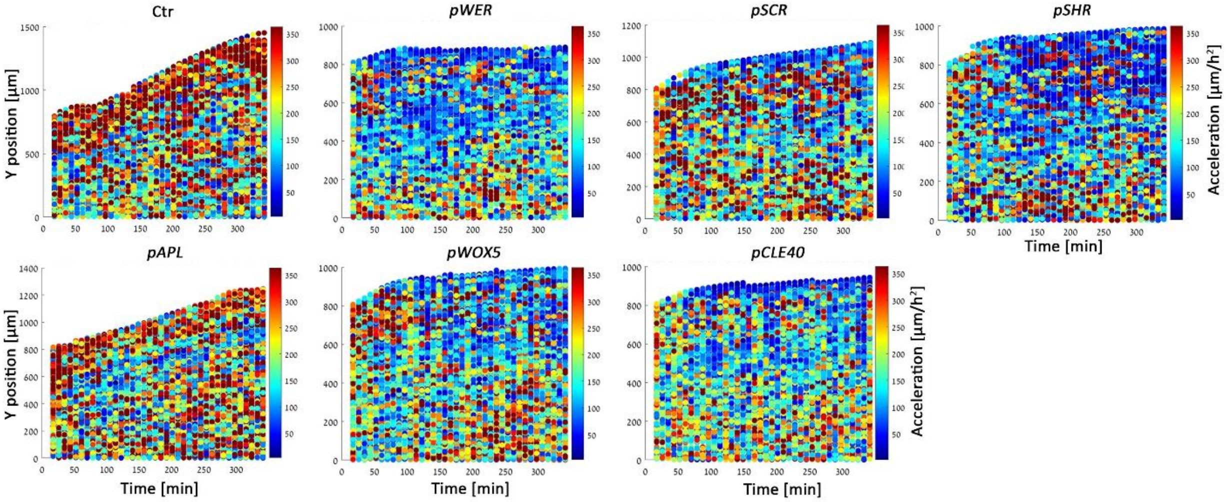

Supplementary Fig. 11. Single-cell acceleration maps following cell-type-specific auxin induction. Control (Ctr) are mock-treated *pWOX5:XVE:YUC1-TAA1; DR5:VENUS; 35S:H2B-RFP* seedlings. Root tip faces upwards. Data are a concatenate of three independent root movies for each genotype, each with ~1000 nuclei. T_0 indicates 20 min following estradiol treatment. On the color scale, red indicates high velocity, and blue indicates low velocity.

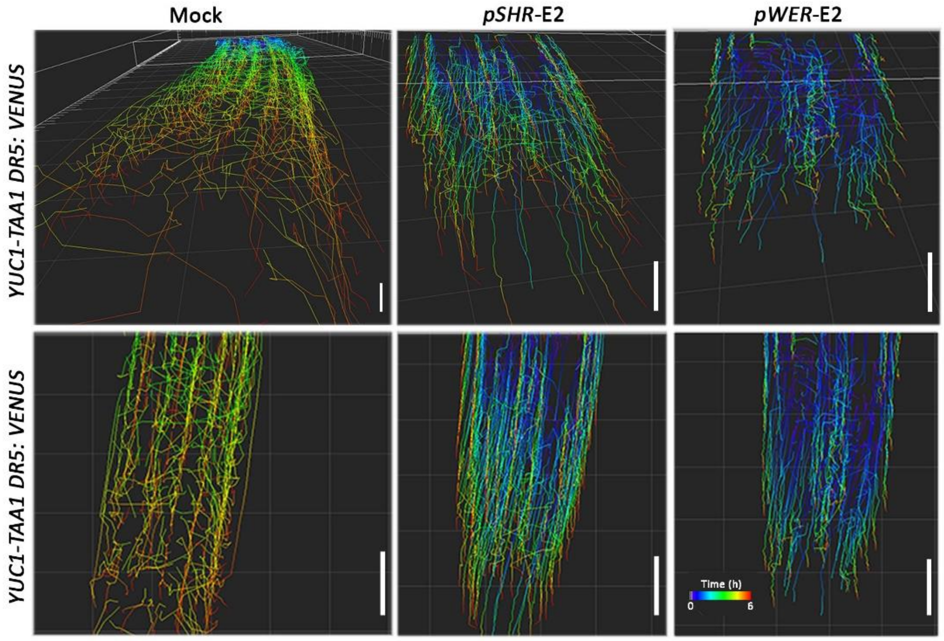

Supplementary Fig. 12. Single-nuclei tracking routes of cell-type-specific *YUC1-TAA1* roots. Shown are cells at the meristem zone cells. Rainbow scale indicates time; T_0 is 20 min following mock or estradiol treatment. "Mock" indicates *pWOX5:YUC1-TAA1; DR5:VENUS* seedlings with no estradiol treatment. Top images are side view; bottom images are top view. Scale bars = 50 µm.

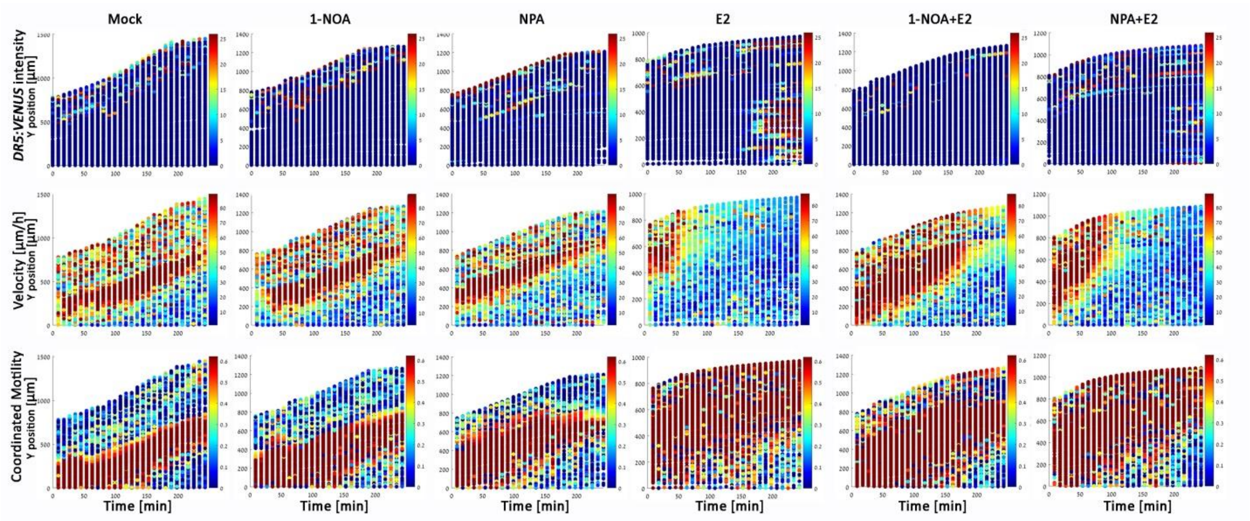

Supplementary Figure 13. 1-NOA and NPA effect on QC-specific auxin-synthesis dependent root growth inhibition and skewing. Single-cell mean *DR5:VENUS* intensity (top), velocity (middle), and coordinated motility (bottom) maps following QC-specific auxin synthesis. *pWOX5:XVE:YUC1-TAA1*; *DR5:VENUS*; *35S:H2B-RFP* seedlings were grown for 4 days on MS agar before transfer to agar containing vehicle (Mock), 50 μM 1-NOA, or 25 μM NPA for 6 h. T_0 indicates 20 min after treatments. Samples were monitored for 4.2 h. Data is from one movies with ~1000 nuclei. The experiment was independently repeated three times.

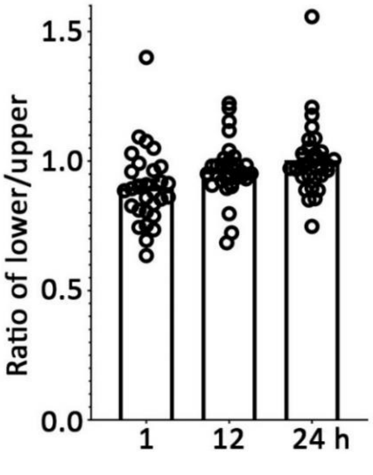

Supplementary Fig. 14. *DR5:VENUS* quantification above and below laser-assisted elimination of stele cells. Shown are means (± SE), n = 29-31 cells from 4 independent experiments for each time point (n = 29 for 1 hour, n = 31 for 12 hours and n = 30 for 24 hours). The differences are not statistically significant, computed from a paired, two-tailed Wilcoxon test, considering the non-normal distribution of the data.

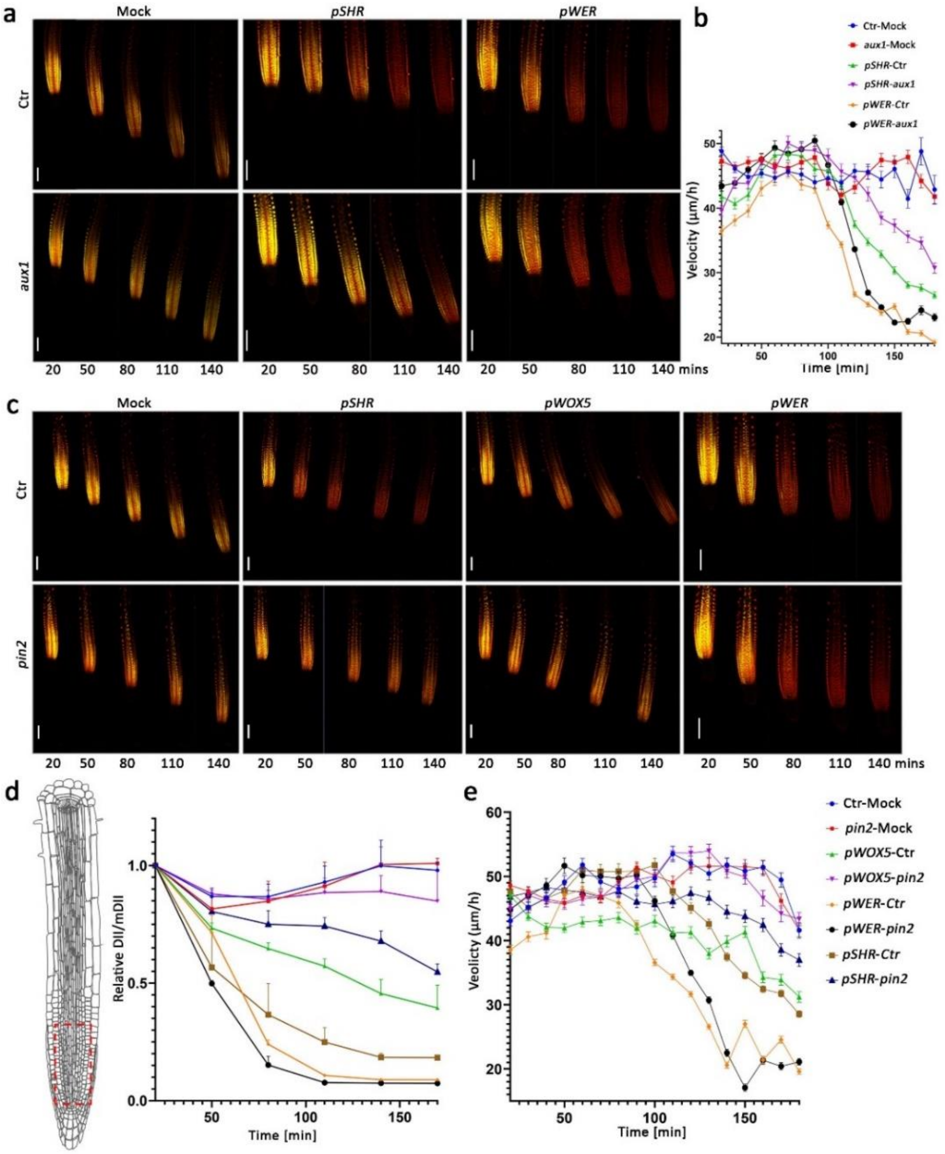

Supplementary Fig. 15. AUX1 and PIN2 are required for auxin basipetal IAA movement and activity. (a) R2D2 response following *YUC1-TAA1* cell-type-specific induction (5 µM estradiol treatment) in the *aux1* background. Mock indicates *pSHR:YUC1-TAA1* plants with no estradiol treatment. Control (Ctr) plants are the same genotype but heterozygous to *aux1*. Presented are merged confocal images of mDII (red) and DII (yellow) The experiment was independently repeated 3 times. Scale bar = 100 µm. **(b)** Quantification of root velocity following 5 µM estradiol treatment of the indicated *aux1*-mutant lines. Data points are concatenated means (± SD) of ~1000 nuclei from two independent root movies, n ≥125 nuclei. **(c)** Time-lapse images of DII ratiometric signals in the indicated *YUC-TAA1 R2D2* lines in the *pin2* background. Mock indicates *pWOX5:YUC1-TAA1* plants with no estradiol treatment. Control (Ctr) plants are the same genotype but heterozygous to *pin2*. Presented are merged confocal images of mDII (red) and DII (yellow). Scale bar = 100 µm. **(d)** Quantification of root relative DII/mDII ratio following 5 µM estradiol and mock treatments of indicated *pin2*-mutant lines. The red box indicates the region used to quantify the DII/mDII ratio. Shown are means (± SE) of two independent roots. **(e)** Quantification of root velocity following 5 µM estradiol treatment of the indicated *pin2*-mutant lines. Data points are concatenated means (± SD) of more than 300 nuclei from two independent root movies. For all experiments, the n number for each line and time point is indicated at Supplementary Source data file.

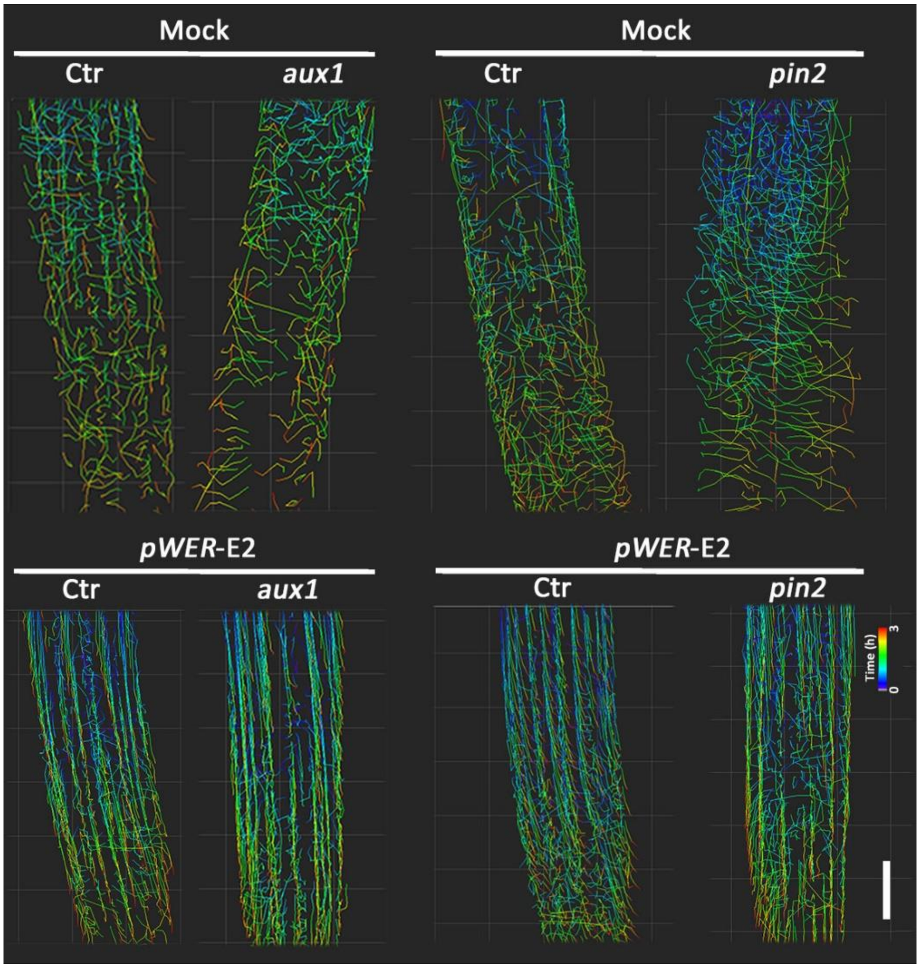

Supplementary Fig. 16. Single-nuclei tracking routes of cells positioned at the meristem zone. Rainbow scale indicates time; T_0 is 10 min following mock or estradiol treatment. For *aux1*, mock-treated plants are *pSHR:YUC1-TAA1*. Control (Ctr) plants are the same genotype but heterozygous for *aux1* (heterozygous lines for promoter-specific *XVE:YUC1-TAA1; R2D2* and *aux1*). *aux1* lines are also heterozygous for promoter-specific *XVE:YUC1-TAA1; R2D2* but homozygous for the *aux1* mutation. For *pin2*, mock-treated plants are *pWOX5:YUC1-TAA1* with no estradiol treatment. Control (Ctr) plants are the same genotype but heterozygous for *pin2* (heterozygous lines for promoter-specific *XVE:YUC1-TAA1; R2D2* and *pin2*). *pin2* lines are also heterozygous for promoter-specific *XVE:YUC1-TAA1; R2D2* but homozygous for the *pin2* mutation. The experiment was repeated independently three times. Scale bars = 50 μm.

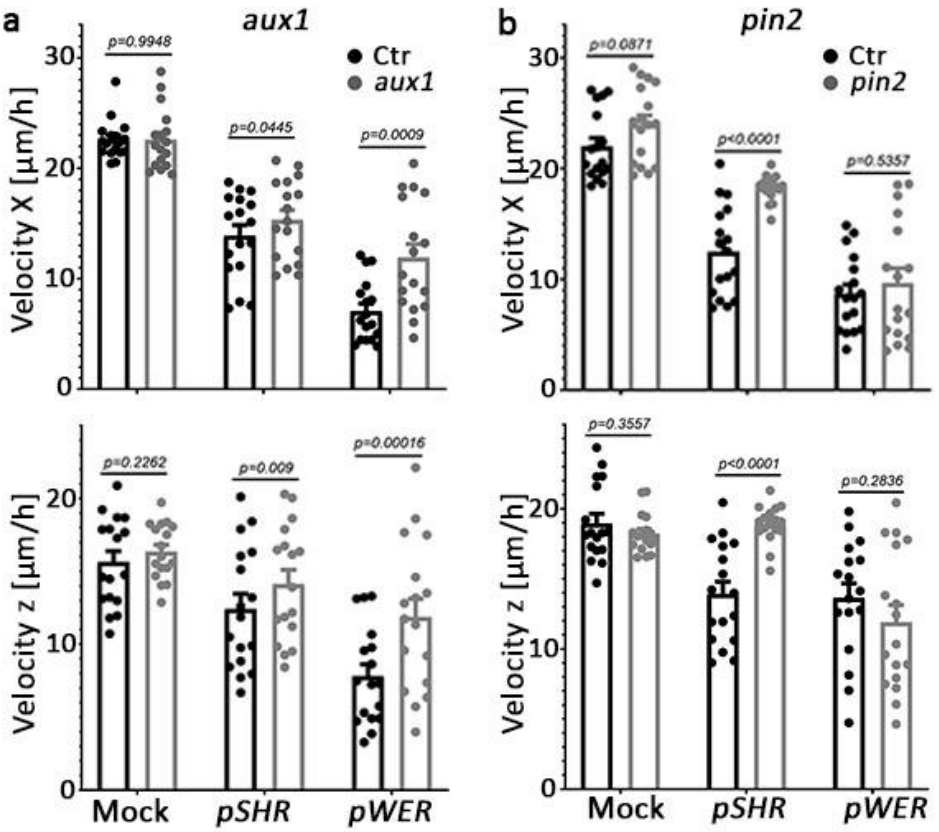

Supplementary Fig. 17. Velocities in the X and Z dimension. Statistical analysis of the single-cell scatters analysis of velocity at the X and Z-dimensions cells shown in Fig. 6i-j. **(a)** For *aux1*, mock-treated plants are *pSHR:YUC1-TAA1* with no estradiol treatment. Control (Ctr) plants are the same genotype but heterozygous for *aux1* (heterozygous lines for promoter-specific *XVE:YUC1-TAA1; R2D2* and *aux1*). a*ux1* lines are also heterozygous for promoter-specific *XVE:YUC1-TAA1; R2D2* but homozygous for the *aux1* mutation. **(b)** For *pin2*, mock-treated plants are *pWOX5:YUC1-TAA1* with no estradiol treatment. Control (Ctr) plants are the same genotype but heterozygous for *pin2* (heterozygous lines for promoter-specific *XVE:YUC1-TAA1; R2D2* and *pin2*). *pin2* lines are also heterozygous for promoter-specific *XVE:YUC1-TAA1; R2D2* but homozygous for the *pin2* mutation.T_0 is 10 min following mock or estradiol treatment. Shown are mean velocities of all time points (± SE) from two independent roots, (n = 17), two-tailed t-test.

Supplementary Table 1: Single-cell morphokinetic parameters. Numbers correspond to Supplementary Fig. 9.

Morphokinetic	Parameter	Description	Equation	No.
Kinetics	Velocity	The magnitude of 2-dimensional velocity vector.	$\lvert V\rvert = \sqrt{V_x^2 + V_y^2 + V_z^2}$	40
	Velocity X	Velocity magnitude in the X axis.	$\vec{V}_x(t) = \frac{\vec{p}_x(t+1) - \vec{p}_x(t-1)}{T(t+1) - T(t-1)}, \quad for\ 1 < t < n$	44
	Velocity Y	Velocity magnitude in the Y axis.	$\vec{V}_y(t) = \frac{\vec{p}_y(t+1) - \vec{p}_y(t-1)}{T(t+1) - T(t-1)}, \quad for\ 1 < t < n$	42
	Velocity Z	Velocity magnitude in the Z axis.	$\vec{V}_z(t) = \frac{\vec{p}_z(t+1) - \vec{p}_z(t-1)}{T(t+1) - T(t-1)}, \quad for\ 1 < t < n$	47
	Acceleration	The magnitude of the 2-dimensional acceleration vector. (Acceleration X is the acceleration magnitude in the X axis and acceleration Y is the acceleration magnitude in the Y axis).	$\vec{a}(t) = \frac{\left(\frac{\vec{p}(t+1) - \vec{p}(t)}{T(t+1) - T(t)} - \frac{\vec{p}(t) - \vec{p}(t-1)}{T(t) - T(t-1)}\right)}{\frac{T(t+1) - T(t-1)}{2}}, \quad for\ 1 < t < n$	45
	Acceleration Z	Acceleration magnitude in the Z axis.	$\vec{a}_z(t) = \frac{\left(\frac{\vec{p}_z(t+1) - \vec{p}_z(t)}{T(t+1) - T(t)} - \frac{\vec{p}_z(t) - \vec{p}_z(t-1)}{T(t) - T(t-1)}\right)}{\frac{T(t+1) - T(t-1)}{2}}, \quad for\ 1 < t < n$	46
	Track displacement length (d_{tot})	The total distance traveled by the particle, the total length of displacements within the track. (Where d (pi, pi+1) is the distance traveled by the nuclei between two adjacent time points).<br>The distance between the first position and last position of a particle along selected axis, the track displacement track in general is the composition of all the axes.	$\lvert d_{tot}\rvert = \sqrt{d_x^2 + d_y^2}$	30
	Track Displacement X	The distance between the first position and the last position of a particle along the X axis.	$d_x = t\overrightarrow{dl} = \lvert\vec{p}_x(N) - \vec{p}_x(1)\rvert$	35
	Track Displacement Y	The distance between the first position and last position of a particle along the Y axis.	$d_y = t\overrightarrow{dl} = \lvert\vec{p}_y(N) - \vec{p}_y(1)\rvert$	29
	Displacement (d_{net})	The shortest distance between first and final positions of the nucleus. It quantifies the distance of the nucleus along a straight line from the initial to the final position.	$d_{\text{net}} = d(\mathbf{p}_1, \mathbf{p}_N)$	32

	Displacement²	The shortest distance between the first time point and time point t position of the nucleus. It quantifies the distance traveled along a straight line from the initial to each time index t position.	$\bar{D}^2(\Delta t) = \sum_{t=t_f+\Delta t}^{t_l} D_x(t,t-\Delta t)^2 + D_y(t,t-\Delta t)^2 + D_z(t,t-\Delta t)^2$ $D_x(t_f,t_l) = P_x(t_l) - P_x(t_f)$ t_L=last time index of track t_F=first time index of track $P_x(t)$=X-position of object at time index t	37
	Confinement Ratio (r_{con})	The ratio between displacement and track length. It is an index, which measures the directional efficiency of the nucleus's track.	$r_{con} = d_{net}/d_{tot}$	1
	Instantaneous Speed (V_i)	The motion rate of a nucleus at a specific time point or moment.	$v_i = d(\mathbf{p}_i, \mathbf{p}_{i+1})/\Delta t$	41
	Instantaneous angle (α_i)	The velocity angle of a nucleus between two adjacent time points.	$\alpha_i = \arctan(y_{i+1} - y_i)/(x_{i+1} - x_i)$	13
	Directional change (γ_i)	The change in direction of a nucleus between two adjacent time points	$\gamma_i = \alpha_i - \alpha_{i-1}$	3
	Mean curvilinear speed	Curvilinear motion is the motion that occurs when a nucleus travels along a curved path. Mean curvilinear speed is the arithmetic mean of the instantaneous speeds.	$\bar{v} = \frac{1}{N-1}\sum_{i=1}^{N-1} v_i$	34
Kinetics	Mean straight-line speed (V_{lin})	The ratio between net distance traveled and the total trajectory time. It is the linear or progressive velocity of the nucleus.	$v_{lin} = d_{net}/t_{tot}$	33
	Linearity of forward progression (r_{lin})	The ratio between mean straight-line speed and mean curvilinear speed. It is an index of efficient progressive velocity of the particle.	$r_{lin} = v_{lin}/\bar{v}$	14

	Collectivity (coll)	An index for collective motility of the nucleus. I) For every time point, all the nuclei within a 200-µm radius from a chosen central cell. II) For every nucleus (j) in the perimeter, the cosines of the velocity angle were calculated in relationship to the chosen central nucleus (i). Results from all nuclei (i) within the entire perimeter (j) were averaged. The coll i (t) is the collectivity of the nuclei i in a certain time point (t).	$coll_{i,j} = \frac{vi_x * vj_x + vi_y * vj_y + vi_z * vj_z}{[(vi_x2 + viy2 + viz2) * (vjx2 + vjy2 + vjz2)]^{1/2}}$ $Coll_i(t) = \frac{1}{N}\sum_{j=1}^{N} coll_{i,j}$	11
Kinetics	Mean squared displacement (MSD)	MSD measures the position deviation over time of a nucleus (relative to a Brownian random motion). A randomly moving nucleus has the same probability to move forward or backward, so the distance (d) traveled by a particle in random motion sums to zero. If, however, instead of adding the distance of each step, the square of the distance is added (d^2), the sum is positive, growing larger with every step. The slope of the MSD is linear at first but when a nucleus's path collides with its neighbors, the MSD will resemble a random walk.<br>The MSD (n) for a given time interval is defined as the average MSD over all independent pairs of points within that time lag, meaning that MSD (1) is the sum of MSDs between adjacent time points, averaged by division to the number of pairs (N-1); MSD (2) is the sum	$\mathrm{MSD}(n) = \frac{1}{N-n}\sum_{i=1}^{N-n} d^2(\mathbf{p}_i; \mathbf{p}_{i+n})$	43

		of MSDs between two-gapped difference time points, averaged by division to the number of pairs (N-2); and MSD (n) = MSD (1) + MSD (2) +…+ MSD (n), while 1<n<N.		
	Full width half maximum (FWHM)	The difference between two time points (t0 and t1) at which the velocity magnitude is equal to half of its maximum (Y0 and Y1, respectively). This feature describes the morphokinetic motility wave.	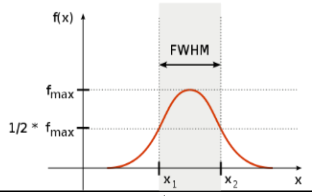	12
	Velocity starting time (t_0)	The first time at which the velocity magnitude is equal to half of its maximum. The time point in which the wave begins.	$t_0(V = \frac{1}{2}V_{max})$	2
	Velocity starting value (Y_0)	The velocity magnitude at velocity starting time (t0). The velocity value at the beginning of the wave.	$V(t_0)$	39
	Velocity ending time (t_1)	The second time at which the velocity magnitude is equal to half of its maximum.	$t_1(V = \frac{1}{2}V_{max})$	48
	Velocity ending value (Y_1)	The velocity magnitude at velocity ending time (t1).	$V(t_1)$	36
	Velocity maximum height	The maximum value of velocity.	V_{max}	38
	Velocity time of maximum height	The time at which maximum height value is achieved.	$t(V_{max})$	49
Morphology	Ellipsoid Axis A X	Vector of the ellipsoid axis a.	$\frac{x^2}{a^2} + \frac{y^2}{b^2} + \frac{z^2}{c^2} = 1$	28
	Ellipsoid Axis A Y			7
	Ellipsoid Axis A Z			4
	Ellipsoid Axis B X	Vector of the ellipsoid axis b.	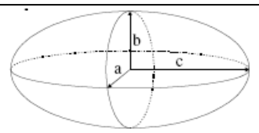	6
	Ellipsoid Axis B Y			10
	Ellipsoid Axis B Z			50
	Ellipsoid Axis C X	Vector of the ellipsoid axis c.	"a", "b" and "c" (the lengths of the three semi-axis) determine the shape of the ellipsoid.	5
	Ellipsoid Axis C Y			51
	Ellipsoid Axis C Z			8

	Ellipticity prolate	If b=c, the shape is a spheroid. If a=b=c, the shape is a perfect sphere. There are two spheroid types: oblate and prolate. A prolate spheroid is cigar-shaped.	$e_{prolate} = \frac{2a^2}{a^2+b^2} * \left(1 - \frac{a^2+b^2}{2c^2}\right)$ 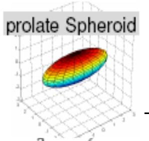	9
	Ellipticity oblate	An oblate spheroid is disk-shaped.	$e_{oblate} = \frac{2b^2}{b^2+c^2} * \left(1 - \frac{2a^2}{b^2+c^2}\right)$ 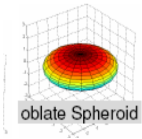	52
	Ellipsoid Axis Length B	Ellipsoid axis length B of a cell.	The lengths of the 'b' semi-axis determine the height of the ellipsoid, see equation in 6-10.	25
	Eccentricity (e)	The fraction of the distance along the semi major axis at which the ellipsoid focus lies. It is a measure of how much the ellipsoid deviates from circular.	$e = \sqrt{1 - \frac{b^2}{a^2}}$	31
	Sphericity	Sphericity is a measure of how spherical the nucleus is. V_p is volume of the particle, A_p is surface of the particle.	$\Psi = \frac{\pi^{\frac{1}{3}}(6V_p)^{\frac{2}{3}}}{A_p}$	20
	Area	The sum of the triangle surfaces. 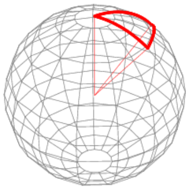 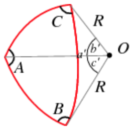 Let a spherical triangle have angles A, B, and C (measured in radians at the vertices along the surface of the sphere) and let the sphere on which the spherical triangle sits have radius R.	$\Delta = R^2[(A+B+C) - \pi]$	27
Fluorescence intens	Intensity Median Ch1	Intensity median of Channel 1 is estimated from the intensity histogram.		22

	Intensity Sum Ch1	Intensity sum of Channel 1 is estimated from the intensity histogram.		26
	Intensity Mean Ch1	Intensity mean of Channel 1 is estimated from the intensity histogram.		23
	Intensity Center Ch1	Intensity center of Channel 1 is estimated from the intensity histogram.		21
	Intensity Max Ch1	Intensity max of Channel 1 is estimated from the intensity histogram.		24
	Intensity Median Ch2	Intensity median of Channel 2 is estimated from the intensity histogram.		15
	Intensity Sum Ch2	Intensity sum of Channel 2 is estimated from the intensity histogram.		16
	Intensity Mean Ch2	Intensity mean of Channel 2 is estimated from the intensity histogram.		17
	Intensity Center Ch2	Intensity center of Channel 2 is estimated from the intensity histogram.		18
	Intensity Max Ch2	Intensity max of Channel 2 is estimated from the intensity histogram.		19

Supplementary Table 2: Primers (cloning and qPCR)

Primers	Sequence (5`-3`)
**Cloning**	
8MWERpF	CAATATATCCTGTCAAACgggagagatgacttcttctgga
8MWERpR	GCCGTTAACGCTTTCATctctttttgtttctttgaatgatagac
8MAPLpF	CAATATATCCTGTCAAACtgtctcatgtgacaatgttttggagga
8MAPLpR	GCCGTTAACGCTTTCATcagaggggattcaagaaacgaatgtga
8MCLE40pF	CAATATATCCTGTCAAACttagttgaagacctccattggt
8MCLE40pR	GCCGTTAACGCTTTCATcttcaaaaacctctttgtggtaag
8MSCRpF	CAATATATCCTGTCAAACattcgaaaaccctaacgactttgga
8MSCRpR	GCCGTTAACGCTTTCATcggagattgaagggttgttggtcgt
8MSHRpF	CAATATATCCTGTCAAACagaagcagagcgtggggtttc
8MSHRpR	GCCGTTAACGCTTTCATcttttaatgaataagaaaatgaatagaagaaaggg
8MWOX5pF	CAATATATCCTGTCAAACaaagacttttatctaccaacttcaa
8MWOX5pR	GCCGTTAACGCTTTCATcgttcagatgtaaagtcctcaactgt
YUC1-CDS-F	CGAATTCACTAGTGATTGGAATGGAGTCTCATCCTCACAAC
YUC1-CDS-R	GGGACGTCGTATGGGTAGGAGGATTTAGAGGTAAAGACAA
TAA1-CDS-F	AAGAGAACCCAGGTCCAAGAATGGTGAAACTGGAGAACTCGAG
TAA1-CDS-R	TCATCGTCCTTGTAGTCAGAAAGGTCAATGCTTTTAATGAGC
**qPCR**	
YUC1-qRT-F	TCAGATCCGGCATGATTCAGATAA
YUC1-qRT-R	TGAAGCCAAGTAGGCACGTT
PP2A-F	TAACGTGGCCAAAATGATGC
PP2A-R	GTTCTCCACAACCGCTTGGT
