# Supplementary material for: Cell kinetics of auxin transport and activity in Arabidopsis root growth and skewing
Source: Nat Commun. 2021 Mar 12;12:1657. doi: 10.1038/s41467-021-21802-3 (PMC7954861; doi:10.1038/s41467-021-21802-3)
Supplement: Supplementary file 3 — Descriptions of Additional Supplementary Files [file 41467_2021_21802_MOESM3_ESM.pdf]

Descriptions of Additional Supplementary Files

Supplementary Movie 1

Description: Root growth in response to cell-type-specific IAA induction. Red: 35S:H2B-RFP (marking nuclei); green: DR5:VENUS. Control (con): pWOX5:XVE:YUC1-TAA1; DR5:VENUS roots without estradiol treatment. Upon estradiol treatment, the indicated promoters express YUC1- 2A-TAA1. T0 is 20 min following estradiol treatment. Scale bar = 200 µm.

Supplementary Movie 2

Description: Single-nuclei image analysis detection in response to cell-type-specific IAA induction. Red: 35S:H2B-RFP (marking nuclei). Control (con): pWOX5:XVE:YUC1-TAA1; DR5:VENUS roots without estradiol treatment. Upon estradiol treatment, the indicated promoters express YUC1-2A-TAA1. T0 is 20 min following estradiol treatment. Scale bar = 200 µm.

Supplementary Movie 3

Description: Tracking of individual nuclei over time and space. Time is indicated by the rainbow scale (0-6 h). Control (con): pWOX5:XVE:YUC1-TAA1; DR5:VENUS roots without estradiol treatment. Upon estradiol treatment, the indicated promoters express YUC1-2A-TAA1. T0 is 20 min following estradiol treatment. Scale bar = 200 µm.

Supplementary Movie 4

Description: Monitoring auxin-dependent root skewing dynamics using long-term vertical-stage microscopy of UV laser ablation sites(19 h). Col-0 Mock-treated (left) 1 µm NAA-treated (right) roots are shown. Time in hours is shown at the top. Black spots indicate for laser ablated cells. Scale bar = 25 µm.

Supplementary Movie 5

Description: Monitoring auxin-dependent root skewing dynamics using long-term vertical-stage microscopy of UV laser ablation sites(25 h). Col-0 Mock-treated (left) 1 µm NAA-treated (right) roots are shown. Time in hours is shown at the top. Black spots indicate for laser ablated cells. Scale bar = 33 µm.

Supplementary Movie 6

Description: Single-nuclei tracking routes of meristem and elongation zones. Shown are cells at the meristem zone (MZ) and elongation zone (EZ). Rainbow scale indicates time; T0 is 20 min following mock or estradiol treatment. “Mock” indicates pWOX5:YUC1-TAA1; DR5:VENUS seedlings with no estradiol treatment. Time is indicated by the rainbow scale. Scale bars = 50 µm.

Supplementary Movie 7

Description: Tracking cell-type-specific auxin-dependent root skewing using long-term verticalstage microscopy. Control (pWOX5:XVE:YUC1-TAA1); DR5:VENUS roots without estradiol treatment. Upon estradiol treatment, the indicated promoters express YUC1-2A-TAA1.Time from estradiol (E2) treatment is shown at the top. Black spots indicate for laser ablated cells. Scale bar = 33 µm
